# Chemical entity extraction using CRF and an ensemble of extractors

**DOI:** 10.1186/1758-2946-7-S1-S12

**Published:** 2015-01-19

**Authors:** Madian Khabsa, C Lee Giles

**Affiliations:** 1 Computer Science and Engineering, The Pennsylvania State University, University Park, PA, USA; 2 Information Sciences and Technology, The Pennsylvania State University, University Park, PA, USA

**Keywords:** Information Extraction, Chemical Entity Extraction, Ensemble Learning, Conditional Random Field

## Abstract

**Background:**

As we are witnessing a great interest in identifying and extracting chemical entities in academic articles, many approaches have been proposed to solve this problem. In this work we describe a probabilistic framework that allows for the output of multiple information extraction systems to be combined in a systematic way. The identified entities are assigned a probability score that reflects the extractors' confidence, without the need for each individual extractor to generate a probability score. We quantitively compared the performance of multiple chemical tokenizers to measure the effect of tokenization on extraction accuracy. Later, a single Conditional Random Fields (CRF) extractor that utilizes the best performing tokenizer is built using a unique collection of features such as word embeddings and Soundex codes, which, to the best of our knowledge, has not been explored in this context before,

**Results:**

The ensemble of multiple extractors outperforms each extractor's individual performance during the CHEMDNER challenge. When the runs were optimized to favor recall, the ensemble approach achieved the second highest recall on unseen entities. As for the single CRF model with novel features, the extractor achieves an F1 score of 83.3% on the test set, without any post processing or abbreviation matching.

**Conclusions:**

Ensemble information extraction is effective when multiple stand alone extractors are to be used, and produces higher performance than individual off the shelf extractors. The novel features introduced in the single CRF model are sufficient to achieve very competitive F1 score using a simple standalone extractor.

## Background

Automatically extracting information from free text has been of interest in many fields because the task is too labor intensive to be carried by humans at scale. Some of the applications try to identify person names and locations that appear in news articles, extract product information from online retailers website, and identify author and title information in scientific publication. Scientists have their share of interest in information extraction. In the case of chemists, they are interested in identifying chemical entities appearing in scientific publications and internal reports of companies. Identifying mentions of chemical entities is crucial for the follow up task of indexing. Some of the leading companies in the chemical domain employ teams that manually identify all chemical entities appearing in their internal reports. Should identifying chemical entities become feasible automatically with high accuracy, the extraction tasks can be done on faster pace, and cover larger collections of documents.

Many approaches have been proposed to tackle the problem of automatically extracting information from free text. The simplest methods relied on dictionaries, sometimes referred to as *gazetteers*, that are compiled by domain experts. Computer programs would check if the document contains terms that are found in the dictionary; for these terms would be extracted as entities. It is, however, challenging to keep a comprehensive dictionary that contains all the entities of interest. Furthermore, dictionary based approaches can not identify new terms that are not in the dictionary. In other words, they fail to generalize beyond the list of chosen terms.

Rule based systems were later introduced as a step forward from dictionaries. Rule based information extraction systems define a set of rules that *identify *mentions of the entity type of interest. The rules are carefully crafted to capture descriptors of the entity that can be used to describe terms of a given entity type. For example, a rule for identifying chemical formulas might be that the term has no two consecutive small case letters. Rules can be cascaded and joined with dictionary look up where the term is inspected for existence in a given dictionary. Although rules provide a way to generalize beyond dictionaries and identify terms that have not been seen before, it is daunting to keep the rules up to date. In fact, crafting the rules in the first place is a very challenging task in many domains. Efforts were made to craft the rules automatically using Inductive Logic Programming (ILP) [1,2].

Machine learning methods for information extraction became prevalent in the late 1990s and early 2000s with the introduction of Maximum Entropy Markov Models (MEMM) [3] and Conditional Random Fields (CRF) [4]. Both algorithms were applied successfully on information extraction tasks, achieving improvements in precision and recall over existing approaches. It seems as if CRF has taken over other methods for *sequence labeling *(information extraction is an application of sequence labeling, where the sequence is a sentence and the labels are the classes of interest that a word can take on) mainly because it solves the label bias problem [4] that other approaches suffer from, including MEMM. However, in many scenarios CRF models do not outperform MEMM models because the large number of parameters that need to be learned in CRFs might lead to overfitting [5].

In the chemical domain, applications of information extraction include identifying chemical formulas and chemical names appearing in research articles and technical reports. OSCAR3 [6] was one of the early systems that used automatic information extraction to extract chemical names and formulas from free text. After that, many researchers introduced new methods and tools for extracting and indexing chemical entities [7-11].

One of the main challenges in chemical information extraction was the lack of annotated corpus in which mentions of chemical entities within documents are identified by human experts. Building an annotated corpus is expensive, and can be challenging to share as the underlying documents might not be copyright-friendly. Therefore, many research teams resort to creating their own annotated corpus on which machine learning extraction algorithms were trained and tested. This, however, has made fairly comparing extraction approaches and systems challenging because it is hard to isolate the source of improvement as it might have came from the approach itself, the features used in the learning algorithm, or the quality of the training corpus.

This obstacle was mainly addressed in the BioCreative's CHEMDNER task with the release of an annotated corpus of 10,000 PubMed abstracts. The CHEMDNER challenge had two tasks. The first is to identify all chemical formulas and names mentioned within abstracts of selected PubMed records. Formally it is referred to as the Chemical Entity Mention (CEM) task. The second is Chemical Document Indexing (CDI) which requires participants to rank all the unique chemical names and formulas within each document. The 10,000 PubMed abstracts were divided into 3,500 abstracts for training, 3,500 abstracts for development, and 3,000 for testing. For details about the CHEMDNER task, please refer to [12].

We have participated in both tasks of the CHEMDNER challenge by submitting 5 runs for each task [13]. For the challenge, our approach involves an ensemble approach that utilized multiple *off-the-shelf *extractors and allowed for combining their output in a probabilistic fashion. The ensemble framework assigns a probability score to each extracted entity that depends on which extractors have identified or failed to identify a given term as an entity. The individual extractors that were used in the ensemble approach were: OSCAR4 [10,14], ChemSpot [11,15], and a modified version of ChemXSeer formula and name tagger [7,8]. The probability score was used later as a confidence measure that allowed for optimizing the extraction result in respect to either precision or recall. When a balanced cut off point was selected for confidence, the F1 score is optimized. This paper discusses our contribution in the CEM task only. For our experiments and results on CDI please refer to [13].

After the end of the competition we revisited the challenge tasks to investigate potential sources of improvement. We started by studying the effect of tokenization on the accuracy of the extractor. The performance of three prominent tokenizers was studied were it was found that OSCAR4 had the highest accuracy in tokenizing the test set of PubMed abstracts. Later, a new Conditional Random Fields extractor was designed using a unique collection of features that utilizes state of the art word embedding algorithms along with *Soundex *code of each term. Soundex is an algorithm that is usually used by the USA Census Bureau to encode surnames *phonetically*. The generated code contains the first letter of the surname combined with three digits representing the the last name *phonetically *(how do they sound). It is used for matching names with multiple spellings. The rationale here for borrowing this last name matching algorithm is that many chemical names tend to sound similar, albeit being spelled and structured differently. To the best of our knowledge, this is the first work that utilizes *Soundex *code to identify chemical entities.

Using the new extractor and the features, we are able to achieve F1 score of 83.3% on the test dataset without doing any post processing on the tags. In the challenge task, most of the top performing teams carried a post processing step that was optimized for this dataset only. As we are interested in building a *general *extractor, we did not do any post processing to boost the F1 score. The CHEMDNER annotation guideline lists abbreviations to chemical entities as valid entity. However, we did not implement or tackle the problem of identifying abbreviations because we believe it is a different problem that can be solved with existing third party solutions such as [16]. Given that the best performing team obtained an F1 score of 88% using multiple extractors and sophisticated post processing and abbreviation matching algorithms, we believe our system achieves a very competitive F1 score as a standalone system that can be easily used as an API or as a program. The source code has been released on Github: https://github.com/SeerLabs/chemxseer-tagger

## Methods

The Methods is split into two sections. In the first section, the approach used during the competition is described in detail, while the second section describes the new extractor created after the competition which we call ChemXSeer Tagger 2.0.

### Ensemble extraction - pre competition

Our interest was initially in the *Chemical Entity Mention *(CEM) task as it is the prerequisite to the following *Chemical Document Indexing *(CDI) task. We started by running a distribution of ChemXSeer's formula and name extractor [7-9] that is released for the general public on the training and development datasets. The tagger is based on Conditional Random Fields (CRF) [4] models, with additional rules for pre and post processing documents. The *off-the-shelf *ChemXSeer tagger was originally trained on a subset of papers from the *Royal Society of Chemistry *(RSC). The extractor performed well when evaluated on precision, but the mediocre recall ended up penalizing the overall F1 score.

We then explored the use of other open source and free chemical information extraction systems, in particular OSCAR4 [10,14], ChemSpot [11,15], Reflect [17], Whatizit [18], and MiniChem [19], on the *BioCreative *dataset. ChemSpot [11] and OSCAR4 (Originally we used the standard setting of OSCAR4, but experiments with the PubMed setting yielded similar results) [10] were promising since the former's result had high F1 score, and the latter's reported high recall compared to the other extractors. To balance the performance and boost both the precision and the recall, we chose two paths to explore. First, modify ChemXSeer's tagger and retrain it on the corpus at hand since the distribution of vocabulary might be different in the *BioCreative *dataset from the original Royal Society of Chemistry (RSC) articles that were used to train ChemXSeer's CRF. The second was to merge the results from all the aforementioned taggers along with ChemXSeer in a way that would improve recall, while sustaining high levels of precision.

#### Modified ChemXSeer

ChemXSeer utilizes two CRF modules, one for extracting formulas and another for extracting chemical names. We created a new unified CRF extractor for all *chemical entities*. The unified extractor merges features that were used for chemical formulas and chemical names. The used features include

• The word itself

• Character level n-grams

• Prefix, postfix, inclusion of punctuation, has superscript

• Regular expressions to match alphanumeric patterns such as the state of capital letters in the word (starts with, mixed caps, ends with cap), the occurrence of digits

• Dictionary look up against a collection of chemical names, chemical elements, known symbols and abbreviations

We also use a window of size *n *for features of the previous and following n words to be included in each word's features, where *n *is set to 1 or 2 based on the feature. After tokenization with the ChemXSeer chemical tokenizer which is based on Lucene StandardAnalyzer[20], each token is assigned to one of the following classes: {*B*, *I*, *O*}, where *B *denotes a start of chemical entity, I denotes a continuation from the previous entity, and *O *for everything else. Three models are created for the purpose of evaluation, one is trained on the training data, the second is trained on the development dataset, and the third is trained on both training and development datasets.

After tagging the sequence of words in a document, those identified as class *B *or *I *are passed to a chemical entity parser which validates that the token is actually a chemical entity. Also we compile a list of common false positives which we denote to as a blacklist. The list can be found on code repository website. An entity candidate is ignored if it is found in the blacklist.

#### Ensemble extraction

We run all the three extractors, ChemxSeer, OSCAR4, and ChemSpot, on the datasets and combine their output as follows. Let token *t *be identified as a chemical entity by at least one of the extractors where *t *is defined as an offset and length only therefore it can refer to unigram or multi-gram token. Assume we have *n *chemical entity extractors, then we are interested in measuring the probability of *t *being an actual entity given the predictions from the n extractors. That is, we would like to estimate

(1) $$P\left(t=Entity\text{|}E_{1}..E_{n}\right)$$

where *E_i _*is an indicator random variable representing the prediction of chemical entity extractor *i*, *i *∈ {1, *n*}, for the token *t*. This represents a discriminative model that tries to infer t given all the results form *E_1_..E_n_*. Luckily, estimating the probabilities and the final conditional probability is not hard because it follows from the performance of each extractor on the annotated dataset. In the case of a single extractor *i*, we can estimate *P*(*t*|*E_i_*) as the precision of extractor *i *on the annotated corpus. When two extractors are used, *i *and *j*, the conditional probability *P *(*t*|*E_i_E_j_*) can be estimated by computing the precision resulting from intersecting the list of chemical entities identified by extractor *i *and *j*. *P *(*t*|*E_i_E_j_*) is interpreted as the probability of correctly identifying a chemical entity when *both *extractors *i *and *j *identified *t *as a chemical entity. In other words, both extractors are used to identify chemical entities, and the intersection of their output is used to compute the precision, which is the estimated conditional probability. Finally, estimating $P\left(t\text{|}E_{i}{\bar{E}}_{j}\right)$, meaning that extractor *i *has identified *t *as a chemical entity while extractor *j *has not identified it as such, is carried by computing the precision resulting from extractor *i *and not *j*. In other words, it is the precision of an extractor whose output is given by {*x *: *x*∈*i*∧*x *∉ *j*}. This approach is generalized to estimate the probabilities using *n *extractors.

For example, let *CO*_2 _be a token that was identified by ChemSpot and OSCAR4 only where ChemXSeer failed to recognize it as a chemical entity. So *E_chemspot _*= 1, *E_oscar _*= 1, and *E_chemxseer _*= 0. The confidence of the term *CO*_2 _is given by

$$\begin{array}{c} P\left(CO_{2}|E_{chemspot}=1,E_{oscar}=1,E_{chemxseer}=0\right)=Precision\left(Y\right)\\ Y=\left\{x:x\in chemspot\wedge x\in oscar\wedge x\notin chemxseer\right\} \end{array}$$

So *Y *is an extractor whose output results from the intersection between OSCAR4 and ChemSpot, minus ChemXSeer.

Since there are 2*^n ^*possible combination for the output of extractors, we need to estimate 2*^n ^*-- 1 parameters for the probabilistic framework to output probability for every possible combination (note that we do not need to estimate the probability when none of the extractors identifies a token to be a chemical entity). While this scales exponentially, it is actually quite easy and fast to estimate the parameters because the expensive part is the extraction itself, and not merging or intersecting results. Since the extraction is done before hand, estimating the parameters only takes fraction of the time needed for extraction. In our case, we have used 3 taggers only, hence there was 7 parameters to estimate.

Ensemble information extraction has been applied before in many applications, including the CHEMDNER challenge that we participated in. One of the popular methods in using multiple extractors together is to feed the output of one extractor as an input to a second extractor to be used as a feature. This approach is often referred to in the literature to as *stacking *[21,22], where multiple extractors are stacked on top of each other such that the output of of several base learners is used as input for the following layer learner. In CHEMDNER multiple teams applied stacking by using the output of ChemSpot as a feature, with a CRF model comprising the final extractor [23-25]. In this case, stacking introduces a serious limitation as the model parameters of the CRF become highly-dependent on the output from individual extractors resulting in smaller weight being assigned to other important features [5]. Another limitation of stacking appears when the extractors use different tokenizers and do not allow tokenization to be performed outside of the classifier software package. In this case, the tokens are not the same, therefore the receiving extractor cannot benefit from the output of the preceding extractor.

Combining the output of multiple extractors in a probabilistic way has been introduced earlier [26]. The approach used in [26] relies on linear interpolation of the classifier class probabilities. The final probability is a weighted average of the individual classifier probability multiplied by the importance of each classifier. The parameters are estimated using cross validation. When the weights are symmetric, each classifier is given equal vote, and the problem becomes majority voting by the collection of extractors. Our method, on the other hand, estimates the actual probabilities of merging the results which can use certain dependencies between the random variables. This way, probabilities depend on the combination of underlying classifiers that generated the output, and not simply on the number of classifiers that generated that output which is the case in majority voting. Furthermore, we do not assume independence of the extractors. This allows us to capture certain relations like what is the probability of an entity being a chemical entity when only ChemSpot and OSCAR4 recognized it as such, while ChemxSeer failed to? This is more powerful than relying on the conditional probability when any two extractors identified the entity as the case of majority voting.

### ChemXSeer Tagger 2.0 - post competition

After the end of the CHEMDNER challenge, we seek to identify areas of improvements that would enhance the extractor's performance. We start by examining the tokenization process as it is the first step in any information extraction application. We later focus on crafting new set of features that would capture the characteristics of chemical entities. These features are used to build a Conditional Random Fields extractor.

### Tokenization

Tokenization has a significant effect on the performance of any information extraction system as tokenizers provide the tokens on which the extractor operates. However, very little attention was paid to the quality of the tokenizers in the CHEMD- NER challenge. Therefore, a study of the performance of tokenizers is needed to justify using one tokenizer instead of the other. The performance of three prominent tokenizers is studied here by examining how accurately they identify the boundaries of the chemical entities in the test data set of the CHEMDNER task. The test set was chosen instead of the training and the development dataset because the annotations were corroborated by a second annotator.

Each tokenizer, ChemSpot, OSCAR4, and ChemXSeer is run to generate offset and length of each token in the test corpus. The results of the tokenizers are given in Table 1. In the table, Correct refer to the number of chemical entities in the test set that were tokenized correctly by the tokenizer. In other words, the tokenizer correctly identified the offset and the end of the token within the document in accordance to the tags provided in the test set. The *split correct *refers to the number of tokens spanning multiple words where space is the only allowed word separator, that were identified correctly by the tokenizer. Overall, there were 25,351 chemical entities in the test set. OSCAR4 had the highest accuracy rate in tokenizing chemical entities at 87%.

**Table 1:** Accuracy of multiple tokenizers when tested on the chemical entities of the test set Word Cosine Similarity.

Measure	ChemSpot	OSCAR4	ChemXSeer
Correct	17149	20491	17869
Split Correct	2379	1744	3190
Total Correct	19528	22235	21059
Incorrect	5823	3116	4292
Accuracy Percentage	77.03%	87.7%	83.06%

The tokenization accuracy on the chemical entities provide an *upper bound *for the highest possible recall by an extractor using the provided tokenizer without performing any post processing on the identified tokens. So, the highest possible recall of an extractor using OSCAR4 tokenizer would be 87%, unless this extractor uses post processing techniques. This helps in explaining the sources of error and potential areas for improvement when it comes to designing better extractors.

Since OSCAR4 tokenizer had the highest accuracy, we adapt it as the default tokenizer in the ChemXSeer Tagger 2.0. The other two tokenizers are available to the CRF extraction software, but OSCAR4 is the default tokenizer.

### Extractor and features

ChemXSeer Tagger 2.0 uses the Conditional Random Fields implementation provided in Mallet [27] to identify chemical entities in the CHEMDNER corpus.

We train the CRF using a "first-order model", thus each pair of labels and observations is assigned a weight. This configuration captures global state information effectively while at the same time avoid over-fitting. Limited Memory BFGS algorithm is used to train the model. Similar to the CRF model developed before the competition, *BIO *is used to label the words.

As CRF works on the sentence level to identify the true labels of the words within each sentence, *Apache openNLP*[28] is used to detect sentence boundaries. Each sentence is then tokenized using OSCAR4 tokenizer. Later, features are extracted to represent each token in the sentence. The feature classes are described below:

#### Word embeddings

Often in many information extraction applications, new terms will show in the test cases that have not been seen previously in the training dataset. To overcome this challenge, word embedding features are incorporated while building the model. The idea behind word embedding is to assign the words of a chosen corpus into multiple clusters such that all the words belonging to a single cluster are *related *to each other. At test time, the cluster Id of the term is used as a feature allowing the model to link the term to other terms that appeared previously in the training dataset using cluster information. Since the words in each cluster are *related*, a new unseen word that belongs to a given cluster that often contains named entities is likely to be a named entity. Word embeddings can be thought of as a transformation of the term to a finite space where elements from this space have been observed previously during the training phase.

The corpus is usually chosen to be large enough such that large number of terms will be observed. In addition, the corpus needs to be representative of the domain of the documents that contain entities which need to be extracted. For example, to extract people names and location information, a corpus about news articles can be used, while to extract chemical entities, a corpus that is built with chemical documents is needed.

Many approaches have been proposed to observe word embeddings features including *Brown Clustering *[29] and *Word2Vec *[30]. In Brown Clustering unigrams are clustered hierarchically based on the bigrams in which they appear, thus forming a dendogram that is encoded using Hoffman code. At training and testing, the Hoffman code or a prefix of the code are used as features to describe the term. In word2vec each term is transformed into a vector based on the surrounding terms appearing next to it in a predefined window. Neural nets are used to infer the vector space representation of each term. For example, Table 2 shows the list of words whose vector representation is similar to *calcium *when cosine similarly is used as distance measure. Later, the vector representaiton of each word is used to cluster the terms using K-Means. Table 3 lists examples from two clusters. Note that words in cluster 8 are chemical entities.

**Table 2:** Similar words to calcium when sorted by cosine similarity.

Word	Cosine Similarity
Ca_2_	0.838966
Ca	0.692185
Thapsigargin	0.565048
Stores	0.562570
Potassium	0.549055
Magnesium	0.539387

**Table 3:** Example of word embedding clusters.

Term	Cluster Id
Tetralinoleoyl	8
thiophosphocholine	8
Phosphoethanolamines	8
y505f	10
Vav	10
Tsad	10

We run word2vec on a corpus containing 700,000 PubMed abstracts for articles appearing in journals where the journal name had the word *chemistry *in it. The corpus contains 213,030 unique terms, that appear 143,301,537 times. We use the default parameter values of word2vec and choose the number of clusters to be 1000. A hash map between the terms and the cluster Ids is created to be used in training and evaluation. As part of the feature generation, each term will be looked up in the hash map for the value of the cluster Id. If the term has not been seen in the corpus, i.e. does not have a cluster Id, the feature is not set.

#### Soundex features

Soundex is an algorithm that is used by the USA Census Bureau to encode surnames *phonetically*. The generated code contains the first letter of the surname combined with three digits representing the the last name *phonetically *(how do they *sound*). Each digit represents a collection of letters that are phonetically similar. Table 4 shows the letter mapping that is used by the Soundex algorithm implemented at the Census office [31]. If the word contains more than three encodable letters, which is the default Soundex implementation, the remaining letters are ignored.

**Table 4:** Soundex code for English letters.

Soundex Code	Letters
1	B, F, P, V
2	C, G, J, K, Q, S, X, Z
3	D, T
4	L
5	M,N
6	R
No Code	A, E, I, O, U, H, W, Y

Soundex is especially effective in matching names with multiple spellings and overcoming spelling mistakes. Soundex provides a powerful mechanism for matching homophones (words that are pronounced similarly but are written differently). For this reason, we borrow this technique as many chemical names tend *sound *similar, albeit being spelled and structured differently. For example, *carbon, carbonate, carbonic*, and *carbonyl *all have the same Soundex code **C-615**. This transformation helps the extractor in identifying chemical entities that did not appear in the training set, but a phonetically similar entity appeared.

We are not aware of any work that utilizes *Soundex *code to identify chemical entities. In our extractor we use the implementation of *Apache openNLP*, while we set the maximum number of allowed digits at 7. Thus we allow the algorithm to encode more letters than the typical Soundex implementation. At training and evaluation, each term is converted to its Soundex code, and a feature is set for each unique value of the Soundex code.

#### General token derived features

A collection of features are derived from the term itself and its shape. These are the following:

• The word itself and lower case version of the word

• Regular expressions to identify if the term contains digits, starts with a capital letter, all capped, all small, mixed cap and small, ends in a sign, ends in a number, contains dash, starts with a number

• Character level n-grams of length 2, 3, and 4

• Heuristic to identify formulas by setting a feature when no two consecutive characters in the term are small case

• Selected features from neighboring terms

• Whether the term marks beginning of a sentence. This is useful in distinguishing proverbs that are capitalized at the sentence beginning from others that are intentionally capitalized

• NLP features based on *BANNER *[32] including lemmatization, and word class conversion

#### Dictionary look up

Dictionaries are used to generate features corresponding to the existence of a given term, or part of it, in the dictionary. The main dictionary used was *Jochem *[33] which contains more than 1.6 million chemical names that were captured from multiple databases. The chemical entities in Jochem were tokenized using OSCAR4 tokenizer because many of them are multi-term entities that would not match a unigram token, which is the unit of tagging in the CRF model. Dictionary tokenization is necessary to ensure that dictionary look up is effective and to avoid the need for prefix matching with a huge dictionary like that of Jochem.

Beyond Jochem, smaller dictionaries were compiled to capture amino acids and their abbreviations. Another dictionary was used to match common prefixes and postfixes that appear in specific groups of chemicals like organic chemistry. A special dictionary of *boost terms *was used to boost certain terms that were occasionally missed by the extractor. In this extractor, the black list has been dropped.

## Results

In this section the results are presented and discussed for the classifier used during the competition, and the CRF classifier developed after the competition.

### Competition extractor - ensemble approach

The performance of the ensemble extractor is presented on both the CEM and CDI tasks. In the ensemble approach, OSCAR4 was combined with the output of ChemSpot and a modified version of ChemXSeer. While ChemSpot and OSCAR4 were used out of the box and did not make use of the provided training dataset, ChemXSeer was trained and tested on opposite datasets. That is, to test ChemXSeer on the development dataset, the model was built using the training dataset only. The parameters of Equation 1 were found to be close enough whether estimated using training or development datasets. Therefore, the final test dataset used the development estimate probabilities. Table 5 shows the estimated probabilities when conditioned on all the possible values for the extractors outcome.

**Table 5:** Probability of a candidate entity conditioned on possible values of the indicator random variables for each of the three taggers used.

ChemxSeer	OSCAR4	ChemSpot	Probability Estimate on Dev	Probability Estimate on Train
1	0	0	0.252	0.26159

0	1	0	0.089	0.08507

0	0	1	0.249	0.25588

1	1	0	0.82083	0.81755

1	0	1	0.72799	0.67361

0	1	1	0.55869	0.53267

1	1	1	0.93316	0.93386

**Figure 1:**
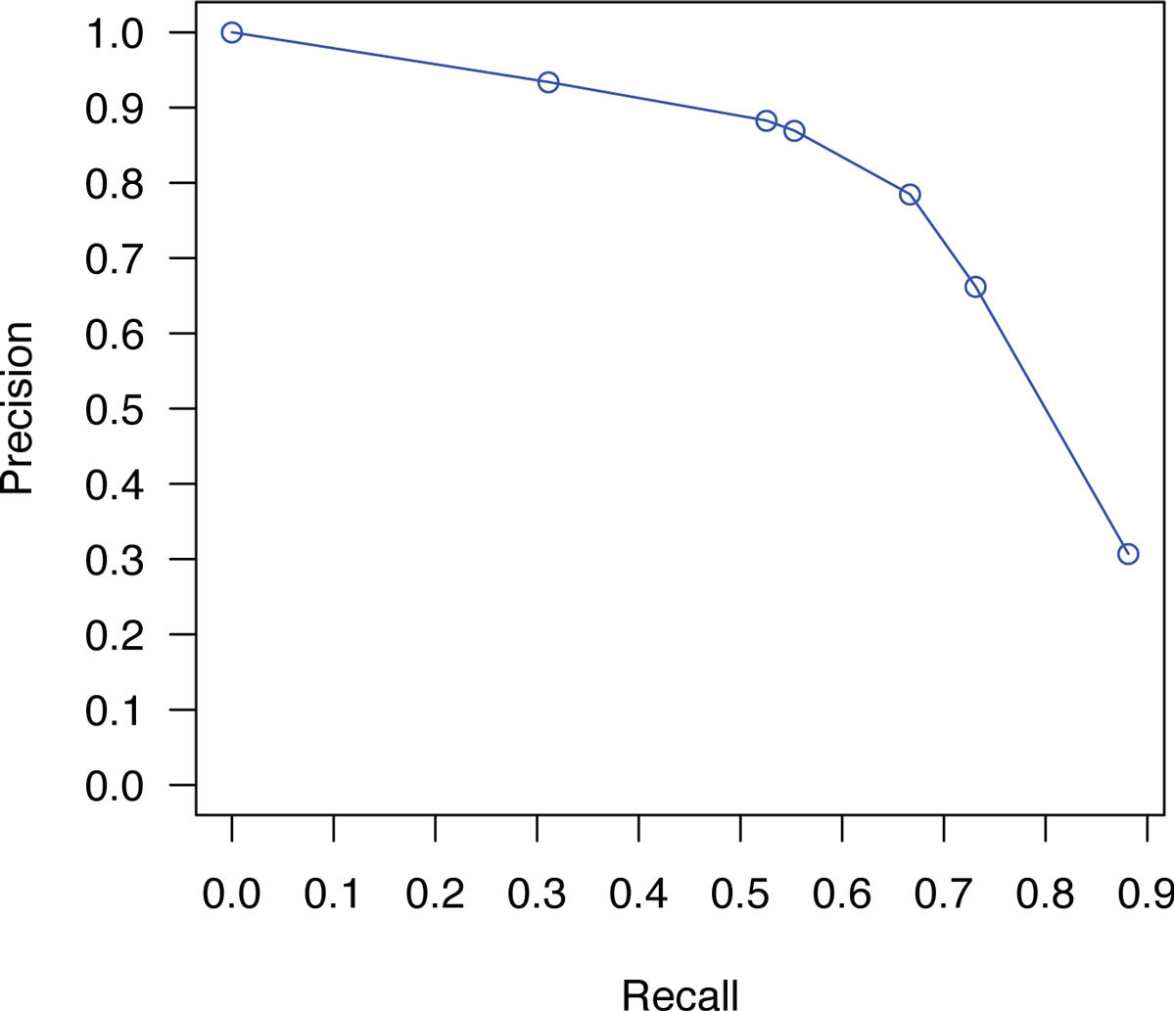
**Precision-Recall curves for CEM task on training dataset**.

**Figure 2:**
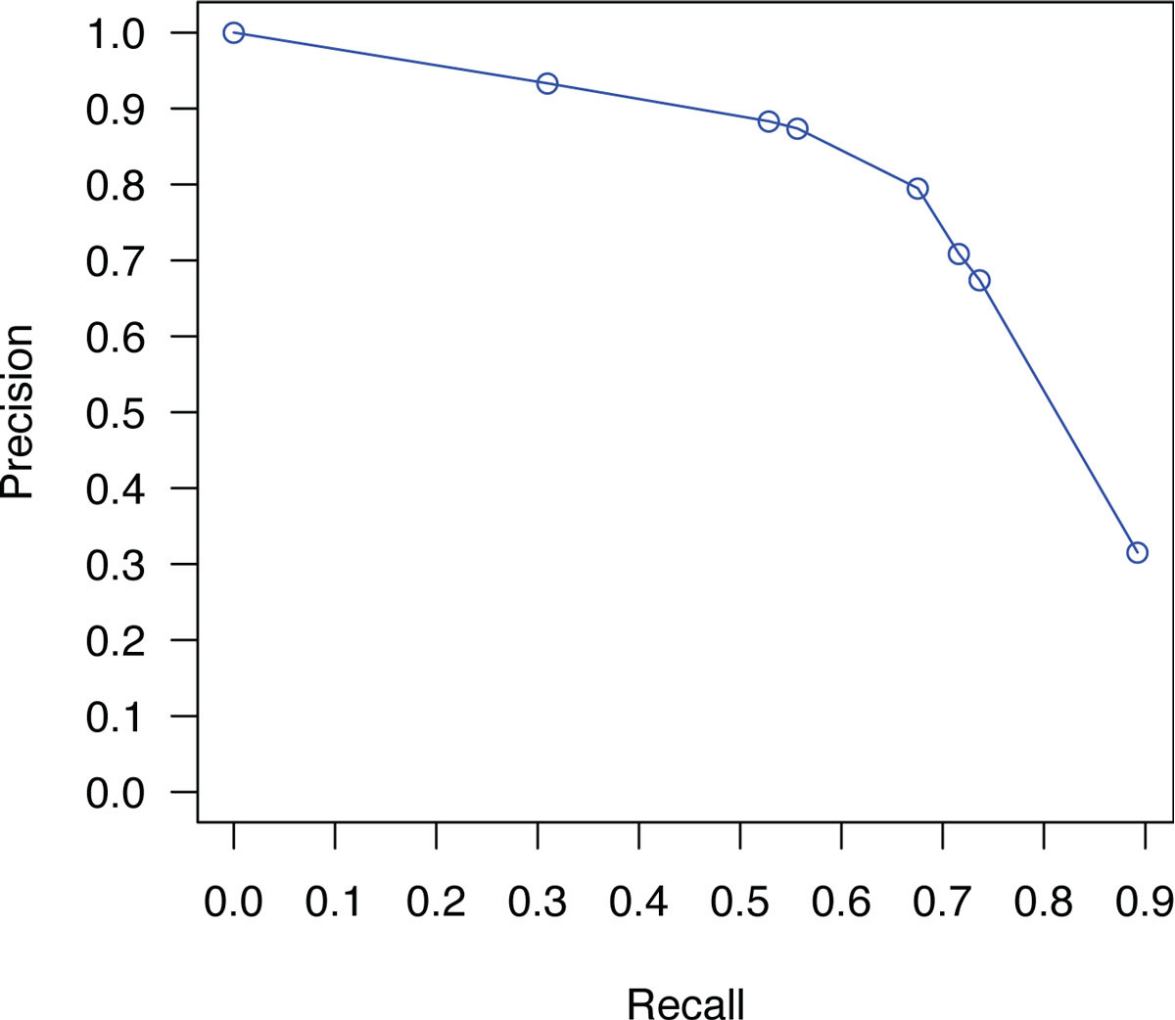
**Precision-Recall curves for CEM task on development dataset**.

Using the probabilities generated from our probabilistic framework, we can apply cut off points based on the confidence assigned to each extracted entity. We have experimented with multiple thresholds and found out that at low threshold values, the recall is favored. The precision is favored over recall as the threshold value increases. Some of the obtained results for the CEM task are summarized in Table 6. The highest obtained F-measure was 73% on the development data, and 72% on training data. Similarly the recall reached a maximum of 89% for development, and 88% for training. Interestingly, one will be able to obtain near 73% recall at 66% of precision. That is, we are able to identify nearly 3/4 of all chemical entities in a document with only 1/3 of these identified entities being false positives. In Figures 1 and 2, we plot precision against various values of recall for the CEM task using both training and development datasets.

**Table 6:** Performance of the ensemble extractor on the CEM task at various confidence thresholds.

Dataset	Threshold	Precision	Recall	F-Measure
Dev	0.01	0.31543	0.8924	0.46611
Dev	0.24	0.67406	0.73650	0.70390
Dev	0.25	0.70871	0.71598	0.71232
Dev	0.5	0.79486	0.67544	0.7303
Dev	0.7	0.87369	0.55663	0.6800
Dev	0.8	0.88315	0.52835	0.66116
Dev	0.9	0.93316	0.30973	0.46509

Train	0.01	0.30711	0.88147	0.45552
Train	0.25	0.66208	0.73126	0.69495
Train	0.26	0.78473	0.66680	0.72098
Train	0.5	0.78473	0.6668	0.72098
Train	0.6	0.86928	0.55312	0.67607
Train	0.7	0.88266	0.52568	0.65893
Train	0.9	0.93386	0.31135	0.467

### Post competition - ChemXSeer Tagger 2.0

The single CRF extractor with the novel feature set was trained using both the training and the development dataset, and tested on the test set. The test was done on the CEM task only, as the extractor was optimized for this task rather than CDI. Multiple runs with different collection of feature set were conducted, and the best performance was obtained using the combination of all the features. The result is shown in Table 7. The F1 score was 83.3%, a significant improvement over previous standalone extractor performance. It is worth mentioning that ChemXSeer Tagger 2.0 does not perform any post processing or abbreviation matching, despite the existence of abbreviations in the dataset. That is because abbreviation matching can be performed by third party tools without the need to complicate the code base.

**Table 7:** Performance of ChemXSeer 2

Run	Precision	Recall	F Score
ChemxSeer Tagger 2.0	0.89569	0.78009	0.83390

The effectiveness of the CRF extractor when multiple set of features are used is shown in Table 8. When word2vec features are removed, the extractor's accuracy drops the most. Soundex features have relatively small contribution in the presence of word2vec features. However, varying the Soundex code length have an effect on the extractor's accuracy as a very short code is not powerful in discriminating chemical entities while a very long code, 100, is counter productive.

**Table 8:** Performance of ChemXSeer 2

Feature	Precision	Recall	F Score
All	0.89730	0.77646	0.83252
All - NLP	0.89749	0.74151	0.81208
All - Word2Vec	0.88993	0.75393	0.81631
All - Soundex	0.89784	0.77342	0.83100
Soundex 3	0.88937	0.76869	0.82464
Soundex 5	0.89647	0.77606	0.83193
Soundex 7	0.89730	0.77646	0.83252
Soundex 100	0.88868	0.76995	0.82507

ChemxSeer Tagger 2.0 has room for improvement. By recalling that the tokenizer had an accuracy of 87%, which is an upper bound on the attainable recall, and comparing that with the 78% recall that ChemxSeer Tagger 2.0 achieved, the tagger can still enhance its performance significantly by identifying the missing 10% of the total entities.

## Conclusion

We introduced an ensemble approach for chemical entity recognition that employs multiple extractors and output probabilities that represent the confidence score for each entity. We showed how these probabilities can be estimated using the training dataset in an effective way. In implementing this approach, we use a modified version of ChemXSeer along with ChemSpot and OSCAR4. Our approach generates probability values that can be used for thresholding each prediction. This aspect can be used to trade-off precision vs. recall. With a higher threshold for probability, our method extracts highly-accurate entities whereas for optimizing recall, a lower threshold on probability can be enforced.

We have also conducted a study about the accuracy of chemical text tokenizers where it was found that OSCAR4 tokenizer outperform others on the CHEMDNER test set. A new extractor, ChemXSeer Tagger 2.0, is built as a CRF extractor that utilizes OSCAR4 tokenizer. We introduced a set of novel features, including word embedding and Soundex that are used in building the CRF extractor which achieves 83.3% F1 score on the test se without any post processing or abbreviation matching. ChemXSeer Tagger 2.0 is designed as an API and can be used as stand alone program. The source code is available on Github: https://github.com/SeerLabs/chemxseer-tagger

## Competing interests

The authors declare that they have no competing interests.

## Authors' contributions

First author had more contribution.
